# The C_4_ Model Grass Setaria Is a Short Day Plant with Secondary Long Day Genetic Regulation

**DOI:** 10.3389/fpls.2017.01062

**Published:** 2017-07-06

**Authors:** Andrew N. Doust, Margarita Mauro-Herrera, John G. Hodge, Jessica Stromski

**Affiliations:** Department of Plant Biology, Ecology and Evolution, Oklahoma State University, StillwaterOK, United States

**Keywords:** Setaria, foxtail millet, green foxtail, photoperiod, short day, long day, flowering time, heading date

## Abstract

The effect of photoperiod (day:night ratio) on flowering time was investigated in the wild species, *Setaria viridis*, and in a set of recombinant inbred lines (RILs) derived from a cross between foxtail millet (*S. italica*) and its wild ancestor green foxtail (*S. viridis*). Photoperiods totaled 24 h, with three trials of 8:16, 12:12 and 16:8 light:dark hour regimes for the RIL population, and these plus 10:14 and 14:10 for the experiments with *S. viridis* alone. The response of *S. viridis* to light intensity as well as photoperiod was assessed by duplicating photoperiods at two light intensities (300 and 600 μmol.m^-2^.s^-1^). In general, day lengths longer than 12 h delayed flowering time, although flowering time was also delayed in shorter day-lengths relative to the 12 h trial, even when daily flux in high intensity conditions exceeded that of the low intensity 12 h trial. Cluster analysis showed that the effect of photoperiod on flowering time differed between sets of RILs, with some being almost photoperiod insensitive and others being delayed with respect to the population as a whole in either short (8 or 12 h light) or long (16 h light) photoperiods. QTL results reveal a similar picture, with several major QTL colocalizing between the 8 and 12 h light trials, but with a partially different set of QTL identified in the 16 h trial. Major candidate genes for these QTL include several members of the PEBP protein family that includes Flowering Locus T (FT) homologs such as OsHd3a, OsRFT1, and ZCN8/12. Thus, Setaria is a short day plant (flowering quickest in short day conditions) whose flowering is delayed by long day lengths in a manner consistent with the responses of most other members of the grass family. However, the QTL results suggest that flowering time under long day conditions uses additional genetic pathways to those used under short day conditions.

## Introduction

Changes in photoperiod are potent signals for plant development, controlling patterns of germination and growth as well as time to flowering and other yield related traits. Photoperiodic response has been studied in many plants, and its manipulation has been of particular importance in the domestication and spread of crops, with a trend toward breeding lines whose flowering times are increasingly photoperiod insensitive (Vergara and Chang, 1985). Decreased photoperiod sensitivity allows crops to be grown in a wider range of environments and increases the utility of elite germplasm. Our knowledge of the effects of photoperiod on flowering time is best developed in *Arabidopsis thaliana*, but much is also known about photoperiodic effects on flowering time in rice and other grasses. We present here a quantitative genetic analysis of flowering time in multiple photoperiod regimes for the panicoid C4 model system Setaria, using a mapping population derived from a cross between domesticated foxtail millet (*S. italica*) and its wild progenitor green foxtail (*S. viridis*).

The effect of photoperiod on flowering time in the grasses has been best studied in rice, where increasing day-length leads to delays in flowering (Lee and An, 2015). There are two photoperiod pathways in rice, one mediated through the CONSTANS ortholog OsHD1, and the other through the grass-specific OsGhd7-OsEhd1 pathway (Nunez and Yamada, 2017). Both OsEhd1 and OsHd1 control homologs of Flowering Locus T (FT), the gene whose protein product is the signal transported from the leaf to the shoot apical meristem to initiate the floral transition. There are two FT homologs in rice, OsHd3a and OsRFT1, the product of a recent tandem duplication (Kojima et al., 2002; Ishikawa et al., 2005; Tamaki et al., 2007; Komiya et al., 2008, 2009). The OsHd1 pathway activates OsHd3a in short days and represses OsHD3a in long days. The OsEhd1 pathway activates OsHd3a in short days and OsRFT1 in long days. Thus, OsHd3a predominantly upregulates flowering in short days while OsRFT1 upregulates flowering in long days. Therefore, even though rice is generally considered a short day plant, it manages to flower in both short days and, eventually, in longer days by two partially distinct genetic pathways (Lee and An, 2015).

The effect of photoperiod on flowering time in other grasses is similar to that of rice, with a general delay of flowering time with longer day-lengths. The pooid grasses are the exception to the general short-day flowering pattern, as most also require vernalization to become competent to flower (Ream et al., 2014; Woods et al., 2016). Thus, the pooid grasses, including Brachypodium, barley and wheat, are long day flowering plants, that natively require winter exposure before becoming competent to flower, although mutations in the vernalization pathway have allowed the production of spring-flowering varieties that have expanded the range of pooid crops (Ream et al., 2014; Woods et al., 2016).

Breeding has in general reduced the photoperiod sensitivity of crops. Breeding for insensitivity was an early step in the improvement of maize, with selection first occurring in pre-historic times by early farmers as they took maize north and south from its site of domestication in central Mexico (Romero Navarro et al., 2017). Similar efforts were made by sorghum breeders in the last century as they moved African tropical germplasm to temperate latitudes in the United States, Australia and Europe (Stephens et al., 1967). Manipulating photoperiod sensitivity is of importance also in the development of new crops such as switchgrass and Miscanthus for biofuels production (Casler et al., 2004, 2007).

The genetic regulation of flowering in panicoid grasses is less well-known than that of rice. Most research has centered on maize and sorghum, which have both been selected for decreased photoperiod sensitivity. However, domestication and improvement history, coupled with the large size and experimentally difficult nature of most panicoid crops has made it difficult to effectively test the effect of different photoperiods in controlled environmental settings. A useful model to test the effects of photoperiod on flowering time in the panicoid grasses is the Setaria system, comprising two species (often considered con-specific), the domesticated cereal foxtail millet (*S. italica*) and its wild progenitor green foxtail (*S. viridis*) (Doust et al., 2009; Li and Brutnell, 2011). The C4 grass genus *Setaria* is a widespread genus in the subfamily Panicoideae, tribe Paniceae, closely related to switchgrass (*Panicum virgatum*) and pearl millet (*Pennisetum glaucum*) within the tribe Paniceae, and to maize (*Zea mays*) and sorghum (*Sorghum bicolor*), in the sister tribe, Andropogoneae (Kellogg et al., 2009; Layton and Kellogg, 2014). Species within the genus have been domesticated several times (Austin, 2006), but the only present-day domesticate is foxtail millet, an ancient grain domesticated in Northern China over 10,000 years ago. Its wild relative, green foxtail, is one of the world’s most widespread weeds (Dekker, 2003), and shows remarkable local adaptation to a wide variety of growing environments (Douglas et al., 1985). One of the more predictable factors that changes over the range of *S. viridis* is photoperiod, with day to night ratios varying between 16 h of daylight at high latitudes to 13 h or even less nearer the equator, making changes in photoperiod likely a primary signal for flowering. Indeed, Setaria has been shown to respond to both small and large changes in photoperiod, and genotypes with very different responses to photoperiod are grown at different latitudes in the various foxtail millet growing regions in China (Li and Yang, 2008; Jia et al., 2013; Diao and Jia, 2017). Accessions of *S. viridis* can be fast-cycling and small-statured, making the system an excellent model for studying photoperiod response in the panicoid grasses.

Flowering time in a Setaria recombinant inbred line (RIL) population has previously been assessed in field, greenhouse, and growth chamber trials (Mauro-Herrera et al., 2013; Doust, 2017), and preliminary studies of flowering time and other traits quantitative trait locus (QTL) analyses have identified several overlapping and several distinct QTL regions, suggesting that flowering time is positively correlated with both height and biomass (Doust, 2017). Here we examine whether flowering time is controlled by different pathways in long and short day regimes in Setaria, using both *S. viridis* and a mapping population between *S. viridis* and its domesticated relative, *S. italica*.

## Materials and Methods

### Plant Materials, Experimental Design, and Phenotyping

Two experiments were performed. In the first, plants of green foxtail (*S. viridis* accession A10.1) were grown in five growth chambers, each set at one of the following photoperiod ratios (light:dark): 8:16, 10:14, 12:12, 14:10 and 16:8. Day and night temperatures were 28°C and 22°C, respectively, and humidity was kept at approximately 30%. Each growth chamber had two shelves, and these were individually adjusted to give an illumination at the level of the soil surface of 600 and 300 μmol.m^-2^.s^-1^, respectively, to investigate the effect of different light fluxes on flowering time. For each combination of photoperiod and light level, there were twelve replicates of *S. viridis* grown in 10 cm × 10 cm × 10 cm square pots and spaced approximately 10 cm apart. Plants were irrigated as needed with an aqueous complete fertilizer mix (Jack’s mix: Nitrogen, Phosphorous and Potassium (20-20-20), JR Peters, Allentown, PA, United States.

In the second experiment, a total of 182 F_7_ RILs from an interspecific cross between *S. italica* accession B100 x *S. viridis* accession A10.1 (Bennetzen et al., 2012), together with their parents, were evaluated for flowering time in a walk-in growth chamber at Oklahoma State University (Stillwater, OK, United States). Three trials were performed, at photoperiod ratios (light:dark) of 8:16, 12:12, and 16:8. The chamber was kept at 30% humidity and day and night temperatures were 28 and 22°C, respectively. The different photoperiod ratios combined with the different day and night temperatures resulted in slightly different average temperatures per trial, with an average of 24°C in the first trial, 25°C in the second trial, and 26°C in the third trial. Illumination from full spectrum fluorescent tubes averaged 200 μmol.m^-2^.s^-1^ at the soil surface, but, owing to the short distance from the light source to the growing plants, actual illumination just before flowering was as much as 400 μmol.m^-2^.s^-1^. Three replicate plants of each RIL were grown in each experiment, with each pot having a single plant. Pots were randomized, and plants were spaced 8.5 cm apart. Pot volume was approximately 215 cm^3^, and pots were filled with Metro-Mix 366 (Sun Gro Horticulture Canada Ltd.). Plants were irrigated as needed with an aqueous complete fertilizer mix (Jack’s mix: Nitrogen, Phosphorous and Potassium (20-20-20), JR Peters, Allentown, PA, United States.

### Phenotypic Measurement

We used days to heading as the measurement of flowering, with plants recorded as flowering when the inflorescence on the main culm was first visible in the sheath of the flag leaf (Mauro-Herrera et al., 2013).

### Statistical Analyses

SPSS version 23 was used to assess the distribution of flowering times for normality. Trait differences between photoperiods were analyzed using analysis of variance (ANOVA). The model fitted for the ANOVA of photoperiod differences between *S. viridis* plants grown at five different photoperiods consisted of two factors, Photoperiod (fixed) and Light Intensity (fixed) and their interaction. The model fitted for the ANOVA of photoperiod differences between the three RIL trials consisted of two factors, Photoperiod (fixed) and RIL (random) and their interaction. η^2^ values were calculated to assess the size of the significant effects of each factor (η^2^ = SS factor/SS total, with η^2^ values summing to 1) (Levine and Hullett, 2002). Bivariate Pearson correlations between photoperiods in the RIL population were examined, and a cluster analysis of the rank order of flowering times of the RILs in the RIL population was conducted using the gplots library in R to detect if rank order remained constant or significantly changed between trials (R-Core-Team, 2016; Warnes et al., 2016).

### QTL Analyses

For QTL analyses we used the previously published 684 marker genetic map (Mauro-Herrera et al., 2013). QTL Cartographer Unix version 1.16 (Basten et al., 1994, 2002) was used for QTL analyses with the composite interval mapping (CIM) method, a genome scan interval of 1 cM, a window size of 10, and the forward and backward regression method (Jansen and Stam, 1994; Zeng, 1994). QTL analyses were conducted for flowering time in each photoperiod trial as well as in a joint analysis. LOD threshold values were estimated via 1000 permutations (Churchill and Doerge, 1994; Doerge and Churchill, 1996).

### Candidate Genes

Candidate genes within identified QTL regions were sought through literature searches, and their position relative to the QTL intervals assessed through comparison with the SNP markers in the map.

## Results

### Differences in Flowering Time between Photoperiods

**FIGURE 1:**
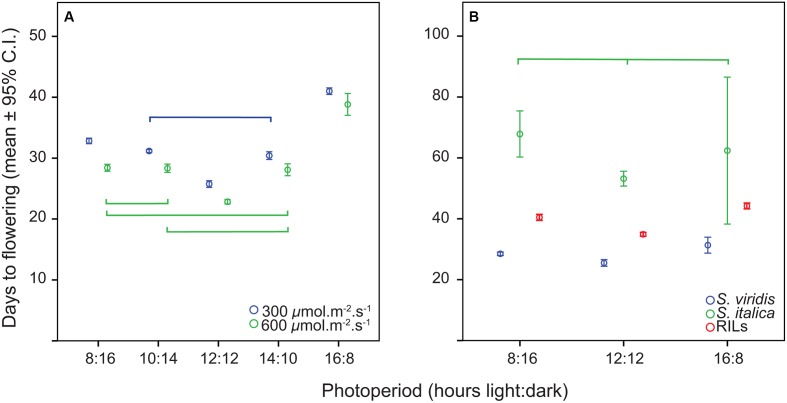
Means and 95% confidence intervals for flowering time in the two trials. **(A)** *S. viridis* photoperiod trial, **(B)** *S. viridis*, *S. italica*, and RILs in QTL trials. Connecting brackets between points indicate which treatments were not significantly different for flowering time, and color indicates to which group the brackets pertain.

The distribution of flowering times in all trials was approximately normal, and therefore all analyses were carried out with the original data. The relationship of flowering time to photoperiod in the first experiment (*S. viridis* growth chamber experiment) was not linear, with plants in the 12 h trial flowering first, followed by 10 and 14 h trials, then 8 h and finally the 16 h trial (**Figure 1A**). In the ANOVA analysis both photoperiod and light level had significant effects on flowering time (**Table 1**), and *post hoc* Tukey HSD analyses on ANOVAs performed at each light level indicate that, for the lower light intensity, all flowering times were significantly different except for the 10 and 14 h light periods. At the higher light intensity, 8 vs. 10, 8 vs. 14, and 10 vs. 14 h light periods were not significantly different. However, at both light levels, the 12 and 16 h photoperiods were significantly different from all others (**Figure 1**). η^2^ values indicate that most of the effect on flowering time was due to differences in photoperiod (87%), and relatively little to differences in light intensity or the interaction between the two factors (**Table 1**).

**Table 1:** Analysis of variance of *Setaria viridis* flowering time differences between the five photoperiod and two light intensities (see Materials and Methods), showing significant effects for both factors and their interaction.

Factor	Sum of squares	df	Mean square	*F*	Sig.	η^2^
Photoperiod	2727.14	4	681.78	482.73	^∗∗∗^	0.87
Light intensity	245.74	1	245.74	174.00	^∗∗∗^	0.08
Photo ^∗^ LI	18.14	4	4.54	3.21	^∗∗^	0.01
Error	148.30	105	1.41			

*The total model explains almost all of the variance (*R*^2^ = 0.952). η^*2*^ values denote the amount of variance explained by each factor and their interaction. Asterisks signify significance level, ^∗∗^*P* < 0.01, ^∗∗∗^*P* < 0.001*.

The relationship of flowering time to photoperiod for *S. viridis* and *S. italica* in the RIL trial (where both parents were included for comparison) was also not linear, with the 12:12 h trial plants flowering first, followed by the 8:16 and 16:8 h trial plants (**Figure 1B**). This was also the case for the mean of the RIL population (**Figure 1B**). However, the “trough” at the 12:12 h photoperiod regime was much less evident than in the growth chamber trials for *S. viridis*, accounting for only a 11% dip between 8 and 12 h as opposed to a 19.5% dip between the same two photoperiod regimes in the growth chamber experiment.

**FIGURE 2:**
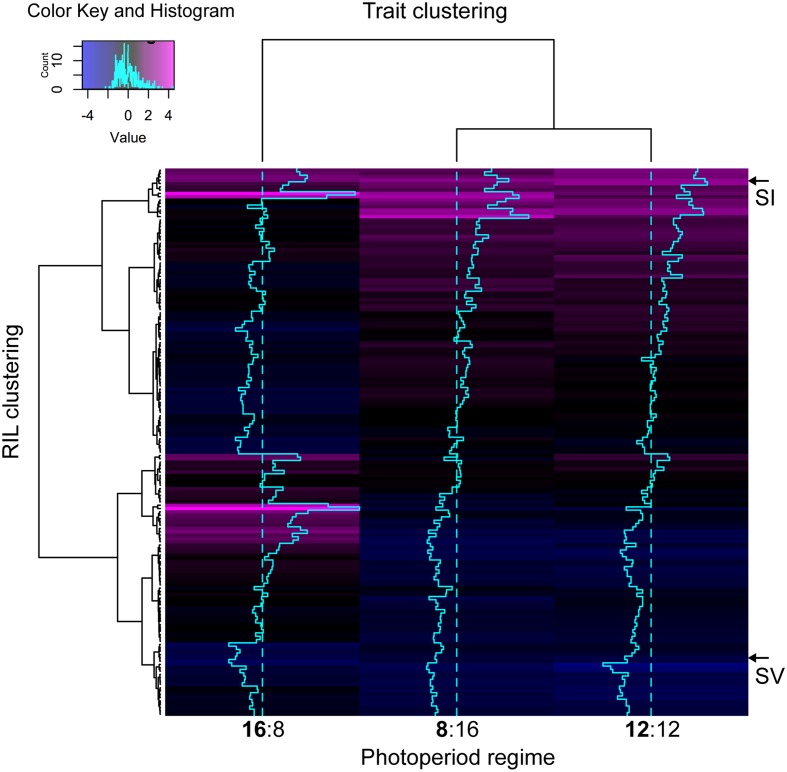
Heatmap visualizing the underlying structure of heading date phenotypes both between individual accessions (rows), and treatment groups (columns). Trace-lines down each treatment group (turquoise) show the degree to which each accession deviates from the mean (dashed line). The A10 *S. viridis* and B100 *S. italica* parents are indicated as ‘SV’ and ‘SI’ respectively. Black or blue coloration indicates that the RIL is early flowering with respect to the rest of the population in that trial, whereas magenta indicates relatively late flowering.

Pearson bivariate correlations of flowering time between photoperiod trials for the RIL population were significant for each pair of trials tested, but explained much more of the variance in the 8 and 12 h trials (*R*^2^ = 0.85) than between the 8 and 16 h (*R*^2^ = 0.04) or the 12 and 16 h (*R*^2^ = 0.05). The cluster analysis of ranked flowering times in the three trials reveals a similar pattern, with different orders of early and late flowering RILs in the 16:8 h trial as compared to the 8:16 and 12:12 h trials (**Figure 2**). In addition, some RILs show the same relative ranking across all three trials (same color in each column of **Figure 2**) whereas others change in ranking across trials, indicating that differents sets of RILs are either photoperiod insensitive or sensitive, respectively.

Analysis of variance of flowering time in the RIL population (with photoperiod as a fixed factor and RIL as a random factor) showed that flowering time differed significantly between photoperiods (**Table 2**). *Post hoc* tests found all flowering times to be significantly different from one another for both *S. viridis* and for the RIL population in all photoperiods. This was not so for *S. italica*, because of the wide spread of flowering times for *S. italica* (**Figure 1B**). The η^2^ values indicate that genotype (RIL) and genotype X photoperiod accounts for most of the variation (50 and 31%, respectively), with photoperiod accounting for only 11%.

**Table 2:** Analysis of variance of the RIL population flowering time differences in the three photoperiod regimes, with Photoperiod (fixed) and RIL genotype (random).

Factor	Type III sum of squares	df	Mean square	*F*	Sig.	η^2^
Photoperiod	23353.71	2	11676.85	65.00^a^	^∗∗∗^	0.11
RIL	104658.49	183	571.90	3.18^b^	^∗∗∗^	0.50
Photo ^∗^ RIL	65617.30	365	179.77	11.67^c^	^∗∗∗^	0.31
Error	17028.53	1105	15.41			

*η^2^ values indicate the relative amount of trait variation explained by each variable. Asterisks signify significance level, ^∗∗∗^*P* < 0.001. Errors for each comparison are as follows: ^a^MS(Photoperiod ^∗^ RIL), ^b^MS(Photoperiod ^∗^ RIL), ^c^MS(Error).η^*2*^ values = SS_*factor*_/SS_*total*_*.

### QTL Analyses

**FIGURE 3:**
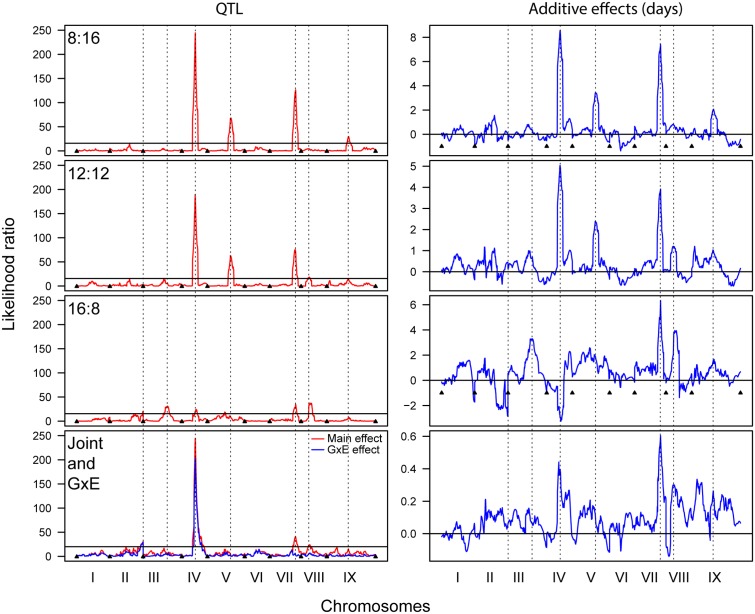
Quantitative trait locus analysis for 8:16, 12:12, and 16:8 photoperiod trials, as well as for a joint analysis showing both main and GxE QTL (Left column), and the corresponding additive effects (Right column). Dashed lines denote significant QTL and indicate whether their positions correspond between trials.

The QTL analyses of flowering time in each photoperiod revealed multiple significant QTL that explained significant proportions of the phenotypic variance (PVE) (**Figure 3** and **Table 3**). The 8 and 12 h trials share a large QTL peak on chromosome IV, whose interval overlaps with a QTL in the 16 h trial. However, the maximum LOD peak is slightly different in the 16 h trial, and the direction of the additive effect is opposite, strongly suggesting that this is not under the same genetic control. The 8 and 12 h trials also share a QTL region on chromosome V that is not found in the 16 h trial. All three trials share a QTL region on chromosome VII with similar additive effects, making this the most stable QTL occurring across trials. The 12 and 16 h trial share a QTL interval on chromosome VIII, and the 8 and 12 h trials share a QTL region on chromosome IX. Finally, the 16 h trial has unique QTL on chromosomes II and III. Almost all of the QTL regions have *S. italica* alleles that increase flowering time, with the only exception being that on chromosome IV in the 16:8 h trial. The joint QTL analysis identified main effect QTL on chromosomes IV, VII, and VIII, and genotype by environment QTL on chromosomes II and IV.

**Table 3:** Position of QTL intervals and percent variation explained (PVE).

Chr.	Start (mbp)	Max (mbp)	Stop (mbp)	PP 8:16 PVE	PP 12:12 PVE	PP 16:8 PVE	Candidate genes	Reference
II	48.4	48.4	49.1			7.6%	*CDF1* (Seita.2G436800),PRR37 (Seita.2G444300)	[Bibr B66][Bibr B49]

III	34.8	41.2	45.5			9.5%	*REF6-like* (Seita.3G307200)	Noh et al., 2004

IV	4.1	12.5	32.8	48.5%	41.8%		*OsCLF* (Seita.4G060900 @4.5)*OsMADS5* (Seita.4G062600 @4.7) *OsMADS55* (Seita.4G077200 @5.96)*FT-like* (Seita.4G102400)@9.1*CONSTANS-like* (Seita.4G116600) @11.4 *Hd3a(OsFTL2)/OsRFT1(OsFTL3)/ZCN15* (Seita.4G122200 @12.5 /Seita.4G067600 @5.2)*HD1/CONSTANS* (Seita.4G122700 @12.55) *OsCRY2* (Seita.4G154000 @20.7) *OsFTL12/ZCN26* (Seita.4G180200 @29.09)	[Bibr B41][Bibr B28]; Jeon et al., 2000 [Bibr B35][Bibr B30]; Tamaki et al., 2007; Komiya et al., 2008, 2009 Yano et al., 2000 Kojima et al., 2002; Tamaki et al., 2007; Komiya et al., 2008, [Bibr B32][Bibr B67][Bibr B20]; Liu et al., 2016 Danilevskaya et al., 2008

IV	8.4	30.7	32.8			9.0% (–)		

V	21.2	27.2	27.9			6.1%	*Hd17/OsELF3* (Seita.5G204600 @26.3)	Matsubara et al., 2008, 2012

V	31.5	35.8	41.9	7.8%	7.8%		*FVE/OsFVE* (Seita.5G291000 @34.8) *OsLFL1* (Seita.5G293900 @35.0)*OsSPA1* (Seita.5G302800 @35.6) *ZCN12-like* (Seita.5G317600 @36.8)	Ausin et al., 2004; Baek et al., 2008 Peng et al., 2007, 2008 Laubinger et al., 2006 Danilevskaya et al., 2008

VII	31.9	33.0	34.3	17.9%	13.3%	17.7%	*HAP5-like* (Seita.7G275200 @32.5)	Ben-Naim et al., 2006; Wenkel et al., 2006

VIII	1.6	2.2	4.0		2.5%		*OsTFL1/OsRCN1/ZCN1/TFL1* (Seita.8G034000 @ 2.2)*OsPRR59* (Seita.8G040100 @2.9)	Shannon and Meekswagner, 1991; Gustafsonbrown et al., 1994; Nakagawa et al., 2002 Murakami et al., 2003, 2005

VIII	2.2	6.4	9.6			13.9%	*FT-like* (Seita.8G106700.1@6.7)	Kojima et al., 2002; Tamaki et al., 2007; Komiya et al., 2008, 2009

IX	22.2	22.9	36.5	3.0%			*REF6-like* (Seita.9G307800 @35.5)	Noh et al., 2004

*Candidate genes found within each interval are also listed, with gene models from *S. italica* (prefix Seita…)*.

### Candidate Genes

There are many genes associated with flowering time that map to these QTL intervals (**Table 3**). The large QTL interval on chromosome IV has previously been shown to contain homologs of a variety of flowering time genes, including *FT*-like and *CONSTANS*-like genes that are syntenic between maize, sorghum, and Setaria (Mauro-Herrera et al., 2013). The interval on Chromosome V in the 8 and 12 h trials contains the Setaria ortholog of *ZCN12*, an FT-like gene. Other members of the PEBP family to which FT belongs where found on chromosome VIII, and include an FT-like and a TFL-like gene.

## Discussion

This study examined the behavior of *S. viridis* in different photoperiods and light intensities as well as the behavior of *S. viridis* and *S. italica* and an F7 RIL population constructed from a cross between them. The means of all trials (parents and mean of the RIL population) showed a pattern of shortest flowering time at the 12:12 photoperiod, followed by longer flowering times at 8, 10, and 14 h light, and longest flowering times at 16 h of light. This data indicates that Setaria is typical for grasses in being a short day species that is also able to flower at longer daylengths, albeit with a delay in flowering time. However, the shortest flowering time is at 12 rather than 10 or 8 h light, in both the RIL experiment and at both light intensities for the growth chamber experiments with *S. viridis*. The simplest explanation is that the shorter daylengths provided insufficient photon flux for optimal development, delaying flowering as a result of a delay in carbon gain. This may be the case for the RIL trials, where average light levels were lower. However, in the *S. viridis* growth chamber experiments, both the 8 and 10 h daylength trials at high light intensity provided a higher flux than in the 12 h low light trial, and yet the 8 and 10 h trials flowered later. This may suggest that the flowering response is gated by a set of genes that have evolved for the naturally shortest light interval, and that shorter light intervals may hinder their interactions. The ANOVA results indicate that most of the variance in flowering time is explained by the difference in photoperiod and less by the difference in light intensity. However, the fact that light intensity can affect flowering time at all speaks to the necessity for a certain net carbon gain by the plant through photosynthesis before transition to an inflorescence meristem can occur. The interaction of light intensity and photoperiod can most clearly be seen between the high intensity 8 h trial and the low intensity 16 h trial, where both trials averaged almost identical light fluxes per day, and where the mean difference in flowering time of 12.6 days was highly significant.

The ANOVA results for the RIL population show that major portions of the variance for the traits are accounted for by the effect of genotype, as well as the interaction between genotype and photoperiod environment. The effect of genotype was expected, given the significant QTL identified for flowering time in a preliminary analysis of genetic map data associated with the RILs (Doust, 2017). The large amount of variance explained by the genotype by photoperiod interaction suggests that there are subsets of the RILs that react differently to the different photoperiod environments. This is in accord with the cluster analysis, which showed that the order of flowering time within the RILs was very different in the 16 h trial from that in the 8 and 12 h trials. Two main patterns are obvious in the cluster analysis, one of which is relative insensitivity to photoperiod, with accessions having a similar ranking in flowering time in all three trials. The other is photoperiodic sensitivity, where accessions flower early in 8 and 12 h trials and late in the 16 h trial or vice versa. The cluster analysis also identified the *S. italica* parent as being consistently among the RILs with the longest time to flowering in all three photoperiod regimes, suggesting that it is relatively photoperiod insensitive. These results accord with previous field and greenhouse studies of flowering time and vegetative architecture in this RIL population, which found significant genotype by environment interactions, including QTL specific for different photoperiod environments (Mauro-Herrera et al., 2013; Mauro-Herrera and Doust, 2016).

The QTL results support the results of the other statistical analyses but also provide a link between underlying genetic patterns and phenotypic effects. There is only one QTL position, that on chromosome VII, that is shared between all three photoperiods and which has an additive effect of the same sign. For this QTL the *S. italica* allele leads to a 4–6 day delay in flowering. In addition to the QTL on chromosome VII, the 8 and 12 h trials share QTL on chromosomes IV and V, with the *S. italica* allele delaying flowering by 4–8 days and 2–4 days, respectively. The 16 h trial has several unique QTL, including those on chromosomes III and V, where the *S. italica* allele delays flowering by 4 and 2 days, respectively. There is also a QTL on chromosome IV, where the interval overlaps with the large QTL in the 8 and 12 h light trial, but the effect is opposite, with the *S. viridis* allele delaying flowering by approximately 3 days. The QTL peaks for the 16 h trial are not as prominent as for the 8 and 12 h trials, but this is partially a result of the QTL graph being scaled to match the 8 and 12 h trials. In fact, each QTL in the 16 h trial explains between 6 and 17% of the variation in flowering time, with effect sizes of between 2 and 6 days. The joint analyses show both main and GxE effects for the QTL on chromosome IV and main effects for the QTL on chromosomes VII and VIII. There is a QTL with a GxE effect only on chromosome II. The QTL results in general mirror those found in field and greenhouse trials (Mauro-Herrera et al., 2013) but the effect sizes vary, suggesting that the size of the effects are greatly affected by genotype by photoperiod interactions.

The candidate genes identified in the various QTL regions include many known to be involved in flowering time control in other grass species. Of these, the most interesting are those that may control the different responses to short and long day photoperiods. The QTL on chromosome V in the 8 and 12 h trials contains the Setaria ortholog of ZCN12, an FT-like gene that is known to be functional in sorghum (Wolabu et al., 2016). The QTL on chromosome II in the joint analysis contains the Setaria ortholog of CDF1, a repressor of CONSTANS, known to be active in grasses grown under long day conditions (Higgins et al., 2010). However, other FT-like genes do not map to QTL in our mapping population, including an FT-like gene that has been reported to have a large effect on photoperiodic control of flowering in sorghum (Cuevas et al., 2016). The QTL region on chromosome VII, common to all trials contains a distant paralog of HAP5B, that may play a role in regulating CONSTANS expression (Ben-Naim et al., 2006; Wenkel et al., 2006). All of these candidates need functional characterization in Setaria, especially those in the 16:8 h trial, but the different QTL patterns indicate that photoperiodic control of flowering in Setaria, and possibly other panicoid grasses, may utilize additional genetic regulation pathways under long day conditions.

The weakly photoperiod sensitive nature of the domesticated parent and highly photoperiod sensitivity of the wild parent of the RIL population are likely representative of extremes that may be found in many accessions of both foxtail and other under-utilized millets. This is unlike their panicoid relatives, maize and sorghum, where the tropical germplasm is the most sensitive to photoperiod, and where there has been intense selection for photoperiod insensitivity to allow for plant growth and flowering at temperate latitudes. These trends are seen in other grasses, such as maize and wheat, where there appears to be a significant shift in newer varieties to more photoperiod insensitive genotypes, as breeders strive to produce varieties that maintain yield in multiple locations (Vergara and Chang, 1985; Bentley et al., 2011; Montesino-San Martin et al., 2014; Jones et al., 2017). Breeding for photoperiod insensitivity is also a goal for millet improvement, and a detailed understanding of the effects of photoperiod on flowering time and the underlying genetic pathways that control photoperiod effects could accelerate such efforts. Understanding the genetic regulation and phenotypic effects of photoperiod variation will also contribute to the production of less photoperiod sensitive elite varieties of switchgrass and other biofuels grasses. The value of the Setaria system, demonstrated in the analysis presented here, is that photoperiod, light flux, temperature, and other variables affecting growth and reproduction can be manipulated so that the effect of each and their interaction, can be assessed. The identification of sets of RILs within the RIL population that vary in their response to different photoperiods will be key to genetically dissecting these factors, along with further exploration of several of the high impact QTL that have been uncovered.

## Author Contributions

AD designed the study, interpreted data, wrote and revised manuscript. MM-H designed experiment, acquired and interpreted data, revised manuscript. JS acquired and interpreted data, revised manuscript. JH interpreted data, revised manuscript for intellectual content.

## Conflict of Interest Statement

The authors declare that the research was conducted in the absence of any commercial or financial relationships that could be construed as a potential conflict of interest.
